# Independent and concomitant associations of gestational diabetes and maternal obesity to perinatal outcome: A register-based study

**DOI:** 10.1371/journal.pone.0221549

**Published:** 2019-08-29

**Authors:** Hilkka Ijäs, Sanna Koivunen, Tytti Raudaskoski, Eero Kajantie, Mika Gissler, Marja Vääräsmäki

**Affiliations:** 1 PEDEGO Research Unit (Research Unit for Pediatrics, Dermatology, Clinical Genetics, Obstetrics and Gynecology), Medical Research Center Oulu (MRC Oulu), Oulu University Hospital and University of Oulu, Oulu, Finland; 2 THL National Institute for Health and Welfare, Department of Chronic Disease Prevention, Helsinki and Oulu, Finland; 3 Children’s Hospital, University of Helsinki and Helsinki University Hospital, Helsinki, Finland; 4 Department of Clinical and Molecular Medicine, Norwegian University of Science and Technology, Trondheim, Norway; 5 Karolinska Institute, Stockholm, Sweden; Helmholtz Zentrum München, GERMANY

## Abstract

**Aims:**

Gestational diabetes (GDM) is often accompanied by maternal overweight. Our aim was to evaluate the separate and concomitant effects of GDM and maternal overweight/obesity on perinatal outcomes.

**Methods:**

We used the Finnish Medical Birth Register to identify all 24,577 women with a singleton pregnancy who delivered in 2009 in Finland and underwent an oral glucose tolerance test (OGTT). Women were divided into groups according to the result of OGTT (GDM/no GDM) and pre-pregnancy body mass index (BMI): normal weight (≤24.9 kg/m^2^), overweight (25.0–29.9 kg/m^2^), and obese (≥30.0 kg/m^2^). Primary outcomes included macrosomia, caesarean delivery, and treatment at neonatal ward. Normal weight women without GDM constituted the reference group.

**Results:**

Compared to reference group, overweight or obese women without GDM had an increased risk of macrosomia [odds ratio adjusted for age, parity, smoking and socio-economic status (aOR)1.18 (95% CI 1.09–1.28) and 1.50 (95% CI 1.19–1.88)], and caesarean delivery [aORs 1.17 (95% CI 1.07–1.28) and 1.52 (95% CI 1.37–1.69)], respectively. In normal weight GDM women the risk of macrosomia [aOR 1.17 (95% CI 0.85–1.62)] and caesarean delivery [aOR 1.10 (95% CI 0.96–1.27)] was not significantly increased as compared to normal weight women without GDM. GDM increased the risk of treatment at neonatal ward in all BMI categories and maternal obesity without GDM was also a risk factor for treatment at neonatal ward. Interaction p values between BMI and GDM on these outcomes were <0.001.

**Conclusions:**

Maternal overweight and obesity without GDM increased the risk of macrosomia and caesarean delivery when compared to the reference group. These risks were amplified when overweight/obesity was accompanied by GDM. Obesity without GDM was a risk factor for treatment at neonatal ward; GDM increased this risk in all BMI categories. Our results suggest that especially maternal obesity should be considered as a risk factor for adverse pregnancy outcomes and GDM further amplifies this risk.

## Introduction

The incidence of gestational diabetes mellitus (GDM) is increasing together with maternal obesity [1]. For example, In Finland, the proportion of overweight (pre-pregnancy body mass index (BMI) 25.0–29.9 kg /m^2^) and obese (BMI ≥30 kg/m^2^) mothers has substantially increased during the 21^st^ century; the rate of overweight mothers increased from 18.8% to 35.6% and that of obese mothers from 7.5% to 13.2% between 1990 and 2013, respectively [2,3]. The incidence of GDM was 19.0% in 2017 in Finland [3]. The European Perinatal Health Report (2010) stated that the proportion of overweight or obese mothers commonly varies from 27% to 37% in European countries [4]. Obesity complicates both pregnancy and delivery, and it increases the costs of maternal care [5,6].

While maternal obesity is a key risk factor of GDM, it is also an independent predictor of adverse pregnancy outcomes including preeclampsia, primary caesarean delivery, and fetal macrosomia [7]. Maternal obesity has been found to be associated with increased cord C-peptide concentration as a marker of fetal hyperinsulinemia and neonatal adiposity as a marker of macrosomia [8,9]. In addition, it is also a risk factor for childhood obesity and long-term metabolic disorders [10].

GDM is often accompanied by overweight or obesity, and the risk of adverse perinatal outcome is highest when these conditions occur together [7]. Previously, it has been shown that both GDM in normal weight woman and maternal obesity without GDM are independent risk factors for adverse perinatal outcome [11,12]. Using the present population-based register study, we investigated the significance of maternal GDM and overweight or obesity alone as well as both conditions combined on the outcome of pregnancy including the incidence of neonatal macrosomia, caesarean delivery, and the need for treatment at neonatal ward.

## Materials and methods

The present study was based on the Finnish Medical Birth Register (MBR), which includes detailed data of the course and complications of pregnancy and delivery along with the perinatal health of the newborn. All pregnancies resulting in a live birth and stillbirth at a gestational age of 22 weeks or more or weighing 500 g or more are included in the MBR. For each delivery in Finland, a structured form for the MBR is completed by the delivery hospital within seven days of delivery. The data are checked at the MBR, and the hospital will be contacted for information suspected of being incorrect or missing. Data are completed through a linkage to the Population Register Centre on live births and the Statistics of Finland on stillbirths and infant deaths. The MBR is complete after these linkages, and its data quality has been shown to be high [13,14]. Since 2004, the MBR has included information on if an oral glucose tolerance test (OGTT) for both the screening and diagnosis of GDM was performed, if the result was abnormal, and if insulin treatment was initiated during pregnancy. National Institute for Health and Welfare gave their permission to use the confidential health register data in this study. No ethical approval statement is needed in Finland, when using anonymous register data only.

The new Finnish national guidelines launched in 2008 recommend that all women should be screened for GDM, excluding those with a very low risk (primiparous women of normal weight under 25 years of age, without a history of diabetes in first-degree relatives, or multiparous women of normal weight less than 40 years of age, with no previous GDM or macrosomic infants). These guidelines were the same as recommended by the American Diabetes Association at the time of the study. A 2-h 75-g oral OGTT is mainly performed between 24 and 28 gestational weeks. For high-risk groups (prior GDM, BMI ≥35 kg/m^2^, or polycystic ovary syndrome with insulin resistance), OGTT is recommended between 12 and 16 gestational weeks. In the case of a normal result, OGTT should be repeated between 24 and 28 gestational weeks. The cut-off values for venous plasma glucose concentrations were 5.3, 10.0, and 8.6 mmol/l at the baseline after an overnight fast and at 1 and 2 h after the glucose load, respectively. The diagnosis of GDM was set after one or more values equal to or greater than the cut-off value. According to the prevailing national guidelines, after the diagnosis of GDM, women receive dietary and lifestyle counselling and begin self-monitoring of glucose concentrations. Insulin therapy is begun if plasma glucose concentrations repeatedly exceeds the target levels (5.5 mmol/l fasting and/or 7.8 mmol/l at 1h postprandial). The use of oral anti-diabetic agents was occasional and not included in the guidelines [15].

**Fig 1:**
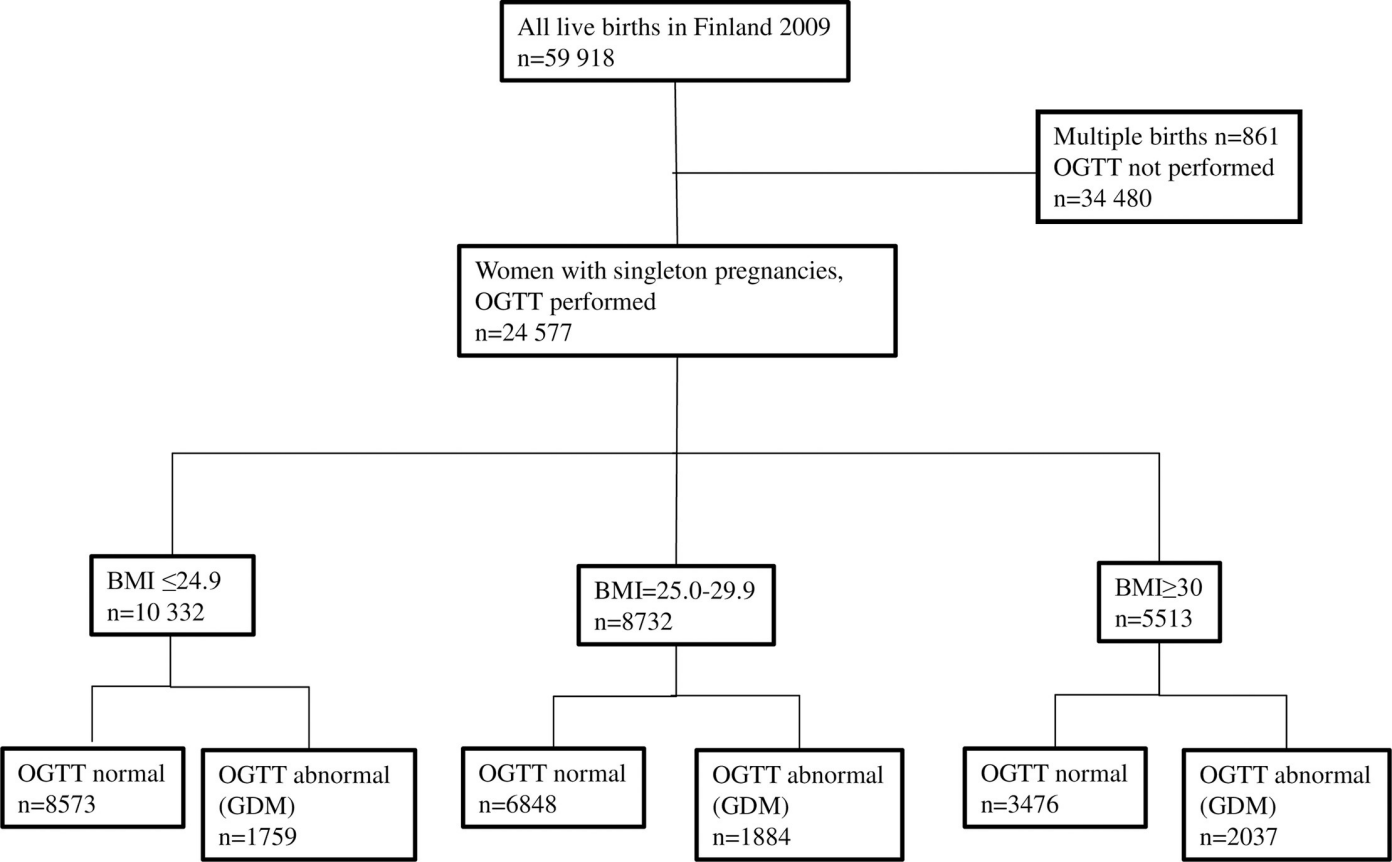
Flowchart.

The study population consisted of women with a singleton pregnancy and OGTT performed during pregnancy (n = 24,577). Those with abnormal OGTT results (n = 5,680, 23.0%) were identified as having GDM, and the others with normal OGTT results acted as controls (n = 18,897, 77.0%). Both groups were further divided into subgroups, according to their self-reported pre-pregnancy BMI, which was recorded at the first antenatal visit: normal weight (BMI ≤24.9 kg/m^2^), overweight (BMI 25.0–29.9 kg/m^2^), and obese (BMI ≥30 kg/m^2^). Pre-pregnancy BMI was calculated as an individual’s pre-pregnancy weight divided by the square of her height, as recorded by the MBR. Self-reported smoking, weight and hence BMI is recorded in the first antenatal visit in maternal health care units, which is practically always during the first trimester (mean 10^th^ gestational week). (Fig 1).

Socioeconomic status was based on maternal occupation during pregnancy, and this information is collected from the delivery hospitals with the MBR form. Coding was based on national standards published by Statistics Finland and were divided into four different groups: 1) Upper-level employees with administrative, managerial, professional and related occupations, 2) Lower level employees with administrative and clerical occupations, 3) Manual workers and 4) Others including for example home mothers, students, pensioners, and self-employed persons [16].

The primary outcome included the incidence of macrosomia, which was defined as large-for-gestational-age (birth weight over +2 standard deviation of the mean) by using the Finnish sex- and gestational age-specific values [17], the rate of caesarean deliveries, and treatment in the neonatal ward. The secondary outcomes were the rates of preterm (<37 +0 gestational weeks) delivery, delivery induction, Apgar score at 5 min, neonatal hypoglycemia, which was defined according to the clinical diagnosis set by a pediatrician (ICD-10 code P70.4), and perinatal mortality defined as stillbirth or death within 7 days after birth.

All statistical analyses were performed using the SAS version 9.3. Categorical variables were reported as frequencies (%), and the Pearson’s χ^2^ test was used to compare the difference in proportions. A logistic regression analysis was used to calculate both the odds ratio (OR) and 95% confidence intervals (CI) for the risk of developing outcomes associated with GDM and BMI. The interaction test was performed to calculate the possible interaction between GDM and obesity. Women of normal weight with normal glucose tolerance were used as a reference group. Adjusted odds ratios (aOR) with 95% CIs were calculated to evaluate the independent associations of maternal age and parity as well as smoking and socioeconomic status with outcome variables in each BMI/GDM group. The analysis code is available from: http://doi10.17605/osf.io/cxq54. (S1 File).

## Results

In 2009, there were 59,057 singleton deliveries in Finland. The mean age of the mothers was 30.0 years, and the mean pre-pregnancy BMI was 24.3 kg/m^2^. The rate of primiparity was 42%. The incidence of an abnormal OGTT result was 9.6% in the whole population.

OGTT was performed for 24,577 (42%) mothers, and GDM was diagnosed by an abnormal result in 23% of them (n = 5,680). Of all the women having OGTT performed, 10,332 (42%) were normal weight, 8,732 (36%) were overweight, and 5,513 (22%) were obese. The baseline characteristics of the study population are listed in Table 1.

**Table 1:** The baseline characteristics of the mothers undergone OGTT.

	No GDM	GDM	
	n = 18897	n = 5680	p-value*
**Age (years)**			
<19	310 (1.6)	94 (1.7)	
20–29	8780 (46.5)	2142 (37.7)	<0.001
30–39	8899 (47.1)	2991 (52.6)	
≥40	908 (4.8)	453 (8.0)	
**Parity**			
Primiparous	8762 (46.4)	2101 (37.0)	<0.001
Multiparous	10 171 (53.6)	3579 (63.0)	
SES*			
I Upper level employees	3345 (17.7)	874 (15.4)	
II Lower level employees	6579 (34.8)	1952 (34.4)	<0.001
III Manual workers	2452 (13.0)	865 (15.2)	
IV Others	6521 (34.5)	1989 (35.0)	
BMI**			
≤24.9 kg/m^2^	8573 (45.4)	1759 (31.0)	
25.0–29.9 kg/m^2^	6848 (36.2)	1884 (33.2)	<0.001
≥30 kg/m^2^	3476 (18.4)	2037 (35.9)	
**Smoking**	2769 (14.7)	1056 (18.6)	<0.001

Data are n (%)

*Sosioeconomic status

**Body Mass Index

The incidence of GDM was 17.0% in normal weight, 21.6% in overweight, and 36.9% in obese women (Table 2). Table 2 shows the incidence of primary outcomes in BMI categories in both the GDM and non-GDM-groups. The incidence of macrosomia was not significantly increased in normal weight women with GDM as compared to normal weight women without GDM. The finding remained the same after adjusting for maternal age, parity, smoking, and socioeconomic status (aOR 1.17, 95% CI 0.85–1.62) (Table 3). Both being overweight or obese without GDM were associated with significantly increased risk of macrosomia with aORs of 1.18 (95% CI 1.09–1.28) and 1.50 (95% CI 1.19–1.88), respectively. Concomitant GDM further increased the risk (Table 3).

**Table 2:** The incidence of primary outcomes according to pre-pregnancy BMI and the GDM status.

	BMI ≤24.9 kg/m^2^			BMI 25–29.9 kg/m^2^			BMI ≥30 kg/m^2^		
	n = 10332			n = 8732			n = 5513		
	No GDM	GDM		No GDM	GDM		No GDM	GDM	
n (%)	8573(83.0)	1759(17.0)	p-value*	6848(78.4)	1884(21.6)	p-value*	3476(63.1)	2037(36.9)	p-value*
Macrosomia	199(2.3)	48(2.7)	0.308	193(2.8)	83(4.4)	<0.001	129(3.7)	135(6.6)	<0.001
Caesarean delivery (CS), all	1369(16.0)	308(17.5)	0.110	1143(16.7)	396(21.0)	<0.001	684(19.7)	515(25.3)	<0.001
Elective CS	495(5.8)	114(6.5)	0.25	386(5.6)	162(8.6)	<0.001	250(7.2)	198(9.7)	0.001
Acute CS	874(10.2)	194(11.0)	0.295	757(11.1)	234(12.4)	0.098	434(12.5)	317(15.6)	0.001
Treatment at neonatal ward	676(7.9)	192(10.9)	<0.001	574(8.4)	225(11.9)	<0.001	365(10.5)	319(15.7)	<0.001

Data are n (%)

*comparison within the same BMI category

**Table 3:** The Odds Ratios (OR) and adjusted ORs of the primary outcome according to pre-pregnancy BMI and the GDM status.

	BMI ≤24.9 kg/m^2^			BMI 25–29.9 kg/m^2^				BMI ≥30 kg/m^2^			
	n = 10332			n = 8732				n = 5513			
	No GDM	GDM		No GDM		GDM		No GDM		GDM	
n (%)	8573 (83.0)	1759 (17.0)		6848 (78.4)		1884 (21.6)		3476 (63.1)		2037 (36.9)	
	OR	OR	aOR	OR	aOR	OR	aOR	OR	aOR	OR	aOR
Macrosomia	1	1.18 (0.86–1.62)	1.17 (0.85–1.62)	1.22 (1.00–1.49)	1.18 (1.09–1.28)	1.94 (1.49–2.52)	1.79 (1.38–2.33)	1.62 (1.30–2.03)	1.50 (1.19–1.88)	2.99 (2.39–3.74)	2.72 (2.17–3.42)
Caesarean delivery (CS), all	1	1.12 (0.98–1.28)	1.10 (0.96–1.27)	1.05 (0.97–1.15)	1.17 (1.07–1.28)	1.40 (1.24–1.59)	1.53 (1.34–1.74)	1.29 (1.16–1.43)	1.52 (1.37–1.69)	1.78 (1.59–2.00)	2.04 (1.81–2.30)
Elective CS	1	1.13 (0.92–1.34)	1.06 (0.86–1.31)	0.97 (0.85–1.12)	1.02 (0.89–1.17)	1.54 (1.28–1.85)	1.44 (1.20–1.74)	1.26 (1.08–1.48)	1.36 (1.16–1.59)	1.76 (1.48–2.09)	1.67 (1.40–1.99)
Acute CS	1	1.09 (0.93–1.29)	1.12 (0.95–1.33)	1.09 (0.99–1.21)	1.26 (1.13–1.40)	1.25 (1.07–1.46)	1.49 (1.27–1.74)	1.26 (1.11–1.42)	1.55 (1.37–1.76)	1.62 (1.41–1.87)	2.06 (1.79–2.38)
Treatment at neonatal ward	1	1.43 (1.21–1.69)	1.44 (1.22–1.71)	1.07 (0.95–1.20)	1.11 (0.99–1.25)	1.58 (1.35–1.86)	1.67 (1.42–1.96)	1.37 (1.20–1.56)	1.45 (1.27–1.66)	2.18 (1.89–2.51)	2.32 (2.01–2.68)

Data are OR (95%CI), aOR = OR adjusted for age, parity, smoking and socio-economic status

The risk of caesarean delivery was similar in women of normal weight with or without GDM (Table 2). Both being overweight and obese without GDM associated with an increased risk of caesarean delivery with aORs of 1.17 (95% CI 1.07–1.28) and 1.52 (95%CI 1.37–1.69), respectively (Table 3). GDM substantially increased the risk of caesarean delivery in both overweight and obese women (Table 2), and the adjustment further strengthened the finding (Table 3). The risk of elective caesarean delivery was not increased in overweight women without GDM, aOR 1.02 (95%CI 0.89–1.17), but their risk of acute caesarean delivery was increased, aOR 1.26 (95% CI 1.13–1.40). Overweight with GDM and obesity with or without GDM increased the risk of both elective and acute caesarean deliveries (Table 3).

The need for treatment at neonatal ward was significantly increased in babies of women with GDM in all BMI categories (Table 2), with the aORs being 1.44 (95% CI 1.22–1.71) for those of normal weight, 1.67 (95% CI 1.42–1.96) for those who were overweight, and 2.32 (95% CI 2.01–2.68) for obese women. Obesity, but not being overweight, without GDM was associated with need for treatment at neonatal ward, with the aOR being 1.45 (95% CI 1.27–1.66) (Table 3). Logistic regression was performed by adjusting additionally with neonatal hypoglycemia: the risk of treatment at neonatal ward was still increased in normal weight GDM women, aOR 1.45 (95%CI 1.21–1.75), overweight GDM women, aOR 1.69 (95%CI 1.42–2.01) and obese GDM women, aOR 2.53 (95%CI 2.17–2.96). Obesity, but not overweight without GDM increased the risk of need for treatment at neonatal ward, the corresponding aORs being 1.38 (95%CI 1.21–1.59) and 1.10 (95%CI 0.98–1.24), respectively.

The incidence of secondary outcomes, according to the BMI and OGTT categories, are listed in Table 4, and their ORs and aORs are shown in Table 5. GDM was associated with the risk of delivery inductions in all BMI categories and both overweight and obesity without GDM increased the risk of delivery inductions. The risk of preterm delivery was increased in women with GDM in all BMI categories. GDM in normal weight and obese women, but not in overweight women, was associated with 5min Apgar score below 7. The risk of neonatal hypoglycemia was increased in women with GDM in all BMI categories, and also in obese women without GDM (Table 5).

**Table 4:** The incidence of secondary outcomes according to the pre-pregnancy BMI and the GDM status.

	BMI ≤24.9kg/m^2^			BMI 25–29.9kg/m^2^			BMI ≥30kg/m^2^		
	n = 10332			n = 8732			n = 5513		
	No GDM	GDM		No GDM	GDM		No GDM	GDM	
n (%)	8573(83.0)	1759(17.0)	p-value*	6848(78.4)	1884(21.6)	p-value*	3476(63.1)	2037(36.9)	p-value*
Delivery induction	1430(16.7)	392(22.3)	<0.001	1333(19.5)	499(26.5)	<0.001	895(25.7)	739(36.3)	<0.001
Preterm delivery	313(3.7)	82(4.7)	0.044	221(3.2)	95(5.0)	<0.001	141(4.1)	113(5.7)	0.011
5min Apgar <7	52(0.6)	20(1.1)	0.015	63(0.9)	14(0.7)	0.467	41(1.2)	25(1.2)	0.875
Neonatal hypoglycemia	125(1.5)	224(12.7)	<0.001	110(1.6)	257(13.6)	<0.001	71(2.0)	313(15.4)	<0.001
Perinatal mortality	19(0.2)	4(0.2)	0.963	15(0.2)	8(0.4)	0.123	11(0.3)	7(0.3)	0.864

Data are n (%)

*comparison within the same weight group

**Table 5:** The Odds Ratios (OR) and adjusted ORs of the secondary outcome according to the pre-pregnancy BMI and the GDM status.

	BMI≤24.9kg/m^2^			BMI 25–29.9kg/m^2^				BMI ≥30kg/m^2^			
	n = 10332			n = 8732				n = 5513			
	No GDM	GDM		No GDM		GDM		No GDM		GDM	
n (%)	8573 (83.0)	1759 (17.0)		6848 (78.4)		1884 (21.6)		3476 (63.1)		2037 (36.9)	
	OR	OR	aOR	OR	aOR	OR	aOR	OR	aOR	OR	aOR
Delivery induction	1	1.43 (1.26–1.62)	1.42 (1.25–1.61)	1.21 (1.11–1.31)	1.21 (1.11–1.31)	1.80 (1.60–2.02)	1.77 (1.57–1.99)	1.73 (1.58–1.90)	1.73 (1.57–1.90)	2.84 (2.56–3.16)	2.79 (2.50–3.10)
Preterm delivery	1	1.29 (1.01–1.65)	1.29 (1.00–1.67)	0.88 (0.74–1.05)	0.91 (0.77–1.09)	1.40 (1.11–1.77)	1.45 (1.14–1.83)	1.12 (0.91–1.37)	1.17 (0.96–1.45)	1.55 (1.24–1.93)	1.62 (1.30–2.03)
5min Apgar <7	1	1.88 (1.12–3.16)	1.90 (1.13–3.19)	1.52 (1.05–2.20)	1.58 (1.09–2.28)	1.23 (0.68–2.22)	1.30 (0.72–2.35)	1.96 (1.30–2.95)	2.07 (1.37–3.13)	2.04 (1.26–3.29)	2.20 (1.35–3.56)
Neonatal hypoglycemia	1	9.86 (7.87–12.36)	9.85 (7.86–12.34)	1.10 (0.85–1.43)	1.10 (0.85–1.43)	10.68 (8.57–13.31)	10.65 (8.53–13.29)	1.41 (1.05–1.89)	1.40 (1.05–1.89)	12.27 (9.91–15.19)	12.18 (9.81–15.11)
Perinatal mortality	1	1.03 (0.35–3.02)	0.92 (0.31–2.72)	0.99 (0.50–1.95)	0.97 (0.49–1.92)	1.92 (0.84–4.39)	1.65 (0.71–3.80)	1.43 (0.68–3.01)	1.36 (0.64–2.90)	1.55 (0.65–3.70)	1.28 (0.53–3.10)

Data are OR (95%CI) and aOR (95%CI), aOR = OR adjusted for age, parity, smoking and socio-economic status

The interaction between GDM and BMI was statistically significant for all variables (p<0.001 for macrosomia, caesarean delivery, NICU treatment, delivery induction, preterm delivery and neonatal hypoglycemia, and p = 0.020 for 5 min Apgar <7), except perinatal mortality (p = 0.230).

## Discussion

In this large, population-based study, obese women without GDM had an increased risk of both macrosomia and caesarean delivery, when compared to normal weight women without GDM. Also in overweight women these risks were slightly, but significantly increased. The risks were further amplified when overweight or obesity were accompanied by GDM. The need for treatment at neonatal ward was increased in the babies of women with GDM in all weight categories and in obese women without GDM. Compared to the reference group, the risk of macrosomia and caesarean delivery was not significantly increased in normal weight women counselled for GDM.

The main strength of our study is the large, population-based dataset and a high rate of validity [13,14]. However, despite the introduction of comprehensive GDM screening, only 42% of the mothers underwent OGTT in Finland in 2009. The new screening protocol had been launched one year prior, but our results show that it had not been fully implemented at that time. Thereafter, coverage has increased annually, reaching 66% in 2017, which has led to increase in the incidence of GDM [3]. It has been estimated that approximately 80% of the Finnish pregnant women meet the criteria of GDM screening according to the current guidelines. Our study may thus not be representative of all pregnant women who should have undergone OGTT and therefore, we included only women whose glucose metabolism was tested during pregnancy to increase the reliability of the results. The register-based data did not allow us to evaluate the significance of pregnancy weight gain or maternal glycemic control, both of which are important for outcome estimates [18–20]. Data concerning GDM risk factors like macrosomia and GDM in prior pregnancies or family history of diabetes were not neither available.

The Hyperglycemia and Adverse Pregnancy Outcome (HAPO) study showed a positive association between increasing maternal glycemic concentrations and macrosomia defined by a birth weight above the 90^th^ percentile [18]. The recent results of the HAPO study group revealed that GDM and obesity—both alone and combined—are associated with adverse pregnancy outcomes and increased risk of macrosomia and primary caesarean, even in women of normal weight with GDM [7]. Contrary to the HAPO study, we found the risk of macrosomia and caesarean delivery in normal weight women with GDM to be comparable to normal weight women without GDM. Because no interventions for mild GDM were included in the HAPO study protocol, women did not receive optimal treatment [18]. By contrast, according to the Finnish uniform guidelines, all women with abnormal OGTT results in our study received dietary counseling and began self-monitoring of their blood glucose concentrations as part of their primary care. Women who repeatedly exceeded the target glucose concentrations were given insulin treatment. Our study represents the effect of implementation of the national guidelines in clinical practice and demonstrates that, through treatment and follow-up of GDM, the risk of macrosomia and caesarean delivery in normal weight women with GDM is comparable to those of the background population. The finding is also in line with the study of Landon et al. which revealed that the treatment of mild GDM decreased the rate of macrosomia, caesarean delivery, and hypertensive complications of pregnancy [21].

Recently, several studies have indicated that maternal obesity is an independent risk factor for neonatal macrosomia [7,19,22,23]. In our study, the risk of macrosomia was slightly, but significantly increased in overweight women and significantly increased in obese women without GDM, which is in line with previous studies.

In line with the study of Wahabi et al., our study revealed equal risk of macrosomia and caesarean delivery in women of normal weight with or without GDM [12]. Maternal overweight or obesity without GDM have been shown to increase the risk of caesarean delivery [12,24,25]. The HAPO study revealed an increased risk of caesarean section in obese primiparous women with normal glucose tolerance during pregnancy [7]. Our study demonstrates a 1.2-fold risk for caesarean delivery in overweight and a 1.5-fold risk in obese mothers without GDM. The concomitant GDM further increased the risk in both groups, as was also seen in previous studies [7,25,26].

The babies of GDM mothers had a 1.4- to 2.3-fold risk of being admitted to a neonatal ward. This is consistent with previous literature [18,26]. In a randomized study of intervention versus routine care after the diagnosis of GDM, Crowther et al. found increased rate of neonatal ward admissions in the intervention group [26]. In our study, the babies of GDM mothers had a 2-fold risk of hypoglycemia compared to the babies in the reference group. This may partly be explained by the advice to follow neonatal glycemia; blood glucose concentrations of babies are followed up routinely when the mother is known to have GDM. However, in our study the risk of neonatal ward treatment in the babies of GDM women remained increased after adjustment with neonatal hypoglycemia. The rate of preterm delivery was also increased in overweight and obese women with GDM and the rate of low 5min Apgar score was increased in normal weight and obese women with GDM, both of which may lead to increased need for treatment at neonatal ward.

We found maternal obesity without GDM to be associated with increased risk of treatment at neonatal ward and also hypoglycemia. These findings are in line with the HAPO study, which revealed that maternal obesity was independently associated with fetal hyperinsulinemia and hence an increased risk of neonatal hypoglycemia [7]. Obese pregnant women without a GDM diagnosis are reported to have higher daytime and nocturnal glucose profiles upon continuous glucose monitoring compared to women of normal weight, despite diet modification, which may explain this finding [27]. It is also possible that obese women may become hyperglycemic after the conventional screening period at 28 gestational weeks; some of them may be detected based on glucosuria in routine urine dipstick screening at later antenatal visits and then referred to OGTT, but for many the condition may remain undetected.

According to our knowledge, there is only one previous study available evaluating the interaction effects of GDM and obesity on perinatal outcomes. The study of Hildeń et al. (2019) revealed no interaction effect between GDM and BMI for severe perinatal outcomes such as malformations, perinatal mortality, stillbirth, prematurity, low Apgar score, fetal distress or Erb’s palsy [28]; that study did not asses our primary outcomes. By contrast, we found that while overweight/obesity alone are associated with macrosomia, caesarean delivery, treatment at neonatal ward (obesity only), delivery induction and low Apgar score, GDM amplifies these risks.

In conclusion, we found maternal obesity without GDM to be risk factor for macrosomia, caesarean delivery and treatment at neonatal ward. Concomitant GDM further amplifies the risk of macrosomia and caesarean delivery in overweight and obese women. The risk of macrosomia and caesarean delivery was not significantly increased in counselled normal weight GDM women when compared to normal weight women without GDM, but their risk of neonatal morbidity was increased. Our findings urge vigilance in the follow-up of especially obese women even with normal glucose tolerance. Further, our results underline the importance of weight management among women of fertile age.

## Supporting information

S1 File SAS code. (DOC)
