# Supplementary material for: Independent and concomitant associations of gestational diabetes and maternal obesity to perinatal outcome: A register-based study
Source: PLoS One. 2019 Aug 29;14(8):e0221549. doi: 10.1371/journal.pone.0221549 (PMC6715199; doi:10.1371/journal.pone.0221549)
Supplement: S1 File — (DOC) [file pone.0221549.s001.doc]

libname synre oracle path='tieto3.thl.fi' user='mikag' schema=synre dbprompt=yes;

libname syre 'k:\j-asema\data\syre\';

**proc** **sql**;

create table peristat as

select tilastovuosi, sukup, sikioita, aiti_ika, tupakointitunnus, aiemmatsynnytykset, soseko, jarjestyskirjain, sdiag1, sdiag2, sdiag3, sdiag4, sdiag5, sdiag6, sdiag7, sdiag8, sdiag9, sdiag10,

rdiag1, rdiag2, rdiag3, rdiag4, rdiag5, rdiag6, rdiag7, rdiag8, rdiag9, rdiag10, sokeri_patol, insuliini_aloitettu, synnytys_paltu, kuolleisuus, syntymapaino, kestovkpv, apaino, apituus,

sokeri_tehty, kaynnistys, synnytystapatunnus, syntymapituus, apgar_1min, apgar_5min, teho, valvonta, muusair, napavaltimoph, paanymparys, LAPSEN_LAHTOPVM , ICD10_1 ,

ICD10_2 , ICD10_3 , ICD10_4 , ICD10_5 , ICD10_6 , ICD10_7 , ICD10_8 , ICD10_9 , ICD10_10 , AITI_TULOPVM ,AITI_LAHTOPVM, lapsen_syntymapvm

from synre.syntymarekisteri

where tilastovuosi eq '2009' and sikioita eq **1** ;

**quit**;

**data** peristat2;

set peristat;

alahto=datepart(aiti_lahtopvm);

atulo=datepart(aiti_tulopvm);

llahto=datepart(lapsen_lahtopvm);

ltulo=datepart(lapsen_syntymapvm);

ennen=ltulo-atulo;

jalkeen=alahto-ltulo;

if tilastovuosi eq **.** then delete;

if sikioita ge **2** then delete;

O24=**0**; O240=**0**; O241=**0**; O242=**0**; O243=**0**; O244=**0**; O249=**0**;

if sdiag1 eq 'O240' then O24=**1**; if sdiag2 eq 'O240' then O24=**1**;

if sdiag3 eq 'O240' then O24=**1**; if sdiag4 eq 'O240' then O24=**1**;

if sdiag5 eq 'O240' then O24=**1**; if sdiag6 eq 'O240' then O24=**1**;

if sdiag7 eq 'O240' then O24=**1**; if sdiag8 eq 'O240' then O24=**1**;

if sdiag9 eq 'O240' then O24=**1**; if sdiag10 eq 'O240' then O24=**1**;

if rdiag1 eq 'O240' then O24=**1**; if rdiag2 eq 'O240' then O24=**1**;

if rdiag3 eq 'O240' then O24=**1**; if rdiag4 eq 'O240' then O24=**1**;

if rdiag5 eq 'O240' then O24=**1**; if rdiag6 eq 'O240' then O24=**1**;

if rdiag7 eq 'O240' then O24=**1**; if rdiag8 eq 'O240' then O24=**1**;

if rdiag9 eq 'O240' then O24=**1**; if rdiag10 eq 'O240' then O24=**1**;

if sdiag1 eq 'O241' then O24=**1**; if sdiag2 eq 'O241' then O24=**1**;

if sdiag3 eq 'O241' then O24=**1**; if sdiag4 eq 'O241' then O24=**1**;

if sdiag5 eq 'O241' then O24=**1**; if sdiag6 eq 'O241' then O24=**1**;

if sdiag7 eq 'O241' then O24=**1**; if sdiag8 eq 'O241' then O24=**1**;

if sdiag9 eq 'O241' then O24=**1**; if sdiag10 eq 'O241' then O24=**1**;

if rdiag1 eq 'O241' then O24=**1**; if rdiag2 eq 'O241' then O24=**1**;

if rdiag3 eq 'O241' then O24=**1**; if rdiag4 eq 'O241' then O24=**1**;

if rdiag5 eq 'O241' then O24=**1**; if rdiag6 eq 'O241' then O24=**1**;

if rdiag7 eq 'O241' then O24=**1**; if rdiag8 eq 'O241' then O24=**1**;

if rdiag9 eq 'O241' then O24=**1**; if rdiag10 eq 'O241' then O24=**1**;

if sdiag1 eq 'O242' then O24=**1**; if sdiag2 eq 'O242' then O24=**1**;

if sdiag3 eq 'O242' then O24=**1**; if sdiag4 eq 'O242' then O24=**1**;

if sdiag5 eq 'O242' then O24=**1**; if sdiag6 eq 'O242' then O24=**1**;

if sdiag7 eq 'O242' then O24=**1**; if sdiag8 eq 'O242' then O24=**1**;

if sdiag9 eq 'O242' then O24=**1**; if sdiag10 eq 'O242' then O24=**1**;

if rdiag1 eq 'O242' then O24=**1**; if rdiag2 eq 'O242' then O24=**1**;

if rdiag3 eq 'O242' then O24=**1**; if rdiag4 eq 'O242' then O24=**1**;

if rdiag5 eq 'O242' then O24=**1**; if rdiag6 eq 'O242' then O24=**1**;

if rdiag7 eq 'O242' then O24=**1**; if rdiag8 eq 'O242' then O24=**1**;

if rdiag9 eq 'O242' then O24=**1**; if rdiag10 eq 'O242' then O24=**1**;

if sdiag1 eq 'O243' then O24=**1**; if sdiag2 eq 'O243' then O24=**1**;

if sdiag3 eq 'O243' then O24=**1**; if sdiag4 eq 'O243' then O24=**1**;

if sdiag5 eq 'O243' then O24=**1**; if sdiag6 eq 'O243' then O24=**1**;

if sdiag7 eq 'O243' then O24=**1**; if sdiag8 eq 'O243' then O24=**1**;

if sdiag9 eq 'O243' then O24=**1**; if sdiag10 eq 'O243' then O24=**1**;

if rdiag1 eq 'O243' then O24=**1**; if rdiag2 eq 'O243' then O24=**1**;

if rdiag3 eq 'O243' then O24=**1**; if rdiag4 eq 'O243' then O24=**1**;

if rdiag5 eq 'O243' then O24=**1**; if rdiag6 eq 'O243' then O24=**1**;

if rdiag7 eq 'O243' then O24=**1**; if rdiag8 eq 'O243' then O24=**1**;

if rdiag9 eq 'O243' then O24=**1**; if rdiag10 eq 'O243' then O24=**1**;

if sdiag1 eq 'O244' then O24=**1**; if sdiag2 eq 'O244' then O24=**1**;

if sdiag3 eq 'O244' then O24=**1**; if sdiag4 eq 'O244' then O24=**1**;

if sdiag5 eq 'O244' then O24=**1**; if sdiag6 eq 'O244' then O24=**1**;

if sdiag7 eq 'O244' then O24=**1**; if sdiag8 eq 'O244' then O24=**1**;

if sdiag9 eq 'O244' then O24=**1**; if sdiag10 eq 'O244' then O24=**1**;

if rdiag1 eq 'O244' then O24=**1**; if rdiag2 eq 'O244' then O24=**1**;

if rdiag3 eq 'O244' then O24=**1**; if rdiag4 eq 'O244' then O24=**1**;

if rdiag5 eq 'O244' then O24=**1**; if rdiag6 eq 'O244' then O24=**1**;

if rdiag7 eq 'O244' then O24=**1**; if rdiag8 eq 'O244' then O24=**1**;

if rdiag9 eq 'O244' then O24=**1**; if rdiag10 eq 'O244' then O24=**1**;

if sdiag1 eq 'O249' then O24=**1**; if sdiag2 eq 'O249' then O24=**1**;

if sdiag3 eq 'O249' then O24=**1**; if sdiag4 eq 'O249' then O24=**1**;

if sdiag5 eq 'O249' then O24=**1**; if sdiag6 eq 'O249' then O24=**1**;

if sdiag7 eq 'O249' then O24=**1**; if sdiag8 eq 'O249' then O24=**1**;

if sdiag9 eq 'O249' then O24=**1**; if sdiag10 eq 'O249' then O24=**1**;

if rdiag1 eq 'O249' then O24=**1**; if rdiag2 eq 'O249' then O24=**1**;

if rdiag3 eq 'O249' then O24=**1**; if rdiag4 eq 'O249' then O24=**1**;

if rdiag5 eq 'O249' then O24=**1**; if rdiag6 eq 'O249' then O24=**1**;

if rdiag7 eq 'O249' then O24=**1**; if rdiag8 eq 'O249' then O24=**1**;

if rdiag9 eq 'O249' then O24=**1**; if rdiag10 eq 'O249' then O24=**1**;

if sdiag1 eq 'O240' then O240=**1**; if sdiag2 eq 'O240' then O240=**1**;

if sdiag3 eq 'O240' then O240=**1**; if sdiag4 eq 'O240' then O240=**1**;

if sdiag5 eq 'O240' then O240=**1**; if sdiag6 eq 'O240' then O240=**1**;

if sdiag7 eq 'O240' then O240=**1**; if sdiag8 eq 'O240' then O240=**1**;

if sdiag9 eq 'O240' then O240=**1**; if sdiag10 eq 'O240' then O240=**1**;

if rdiag1 eq 'O240' then O240=**1**; if rdiag2 eq 'O240' then O240=**1**;

if rdiag3 eq 'O240' then O240=**1**; if rdiag4 eq 'O240' then O240=**1**;

if rdiag5 eq 'O240' then O240=**1**; if rdiag6 eq 'O240' then O240=**1**;

if rdiag7 eq 'O240' then O240=**1**; if rdiag8 eq 'O240' then O240=**1**;

if rdiag9 eq 'O240' then O240=**1**; if rdiag10 eq 'O240' then O240=**1**;

if sdiag1 eq 'O241' then O241=**1**; if sdiag2 eq 'O241' then O241=**1**;

if sdiag3 eq 'O241' then O241=**1**; if sdiag4 eq 'O241' then O241=**1**;

if sdiag5 eq 'O241' then O241=**1**; if sdiag6 eq 'O241' then O241=**1**;

if sdiag7 eq 'O241' then O241=**1**; if sdiag8 eq 'O241' then O241=**1**;

if sdiag9 eq 'O241' then O241=**1**; if sdiag10 eq 'O241' then O241=**1**;

if rdiag1 eq 'O241' then O241=**1**; if rdiag2 eq 'O241' then O241=**1**;

if rdiag3 eq 'O241' then O241=**1**; if rdiag4 eq 'O241' then O241=**1**;

if rdiag5 eq 'O241' then O241=**1**; if rdiag6 eq 'O241' then O241=**1**;

if rdiag7 eq 'O241' then O241=**1**; if rdiag8 eq 'O241' then O241=**1**;

if rdiag9 eq 'O241' then O241=**1**; if rdiag10 eq 'O241' then O241=**1**;

if sdiag1 eq 'O242' then O242=**1**; if sdiag2 eq 'O242' then O24=**1**;

if sdiag3 eq 'O242' then O242=**1**; if sdiag4 eq 'O242' then O24=**1**;

if sdiag5 eq 'O242' then O242=**1**; if sdiag6 eq 'O242' then O24=**1**;

if sdiag7 eq 'O242' then O242=**1**; if sdiag8 eq 'O242' then O24=**1**;

if sdiag9 eq 'O242' then O242=**1**; if sdiag10 eq 'O242' then O24=**1**;

if rdiag1 eq 'O242' then O242=**1**; if rdiag2 eq 'O242' then O242=**1**;

if rdiag3 eq 'O242' then O242=**1**; if rdiag4 eq 'O242' then O242=**1**;

if rdiag5 eq 'O242' then O242=**1**; if rdiag6 eq 'O242' then O242=**1**;

if rdiag7 eq 'O242' then O242=**1**; if rdiag8 eq 'O242' then O242=**1**;

if rdiag9 eq 'O242' then O242=**1**; if rdiag10 eq 'O242' then O242=**1**;

if sdiag1 eq 'O243' then O243=**1**; if sdiag2 eq 'O243' then O243=**1**;

if sdiag3 eq 'O243' then O243=**1**; if sdiag4 eq 'O243' then O243=**1**;

if sdiag5 eq 'O243' then O243=**1**; if sdiag6 eq 'O243' then O243=**1**;

if sdiag7 eq 'O243' then O243=**1**; if sdiag8 eq 'O243' then O243=**1**;

if sdiag9 eq 'O243' then O243=**1**; if sdiag10 eq 'O243' then O243=**1**;

if rdiag1 eq 'O243' then O243=**1**; if rdiag2 eq 'O243' then O243=**1**;

if rdiag3 eq 'O243' then O243=**1**; if rdiag4 eq 'O243' then O243=**1**;

if rdiag5 eq 'O243' then O243=**1**; if rdiag6 eq 'O243' then O243=**1**;

if rdiag7 eq 'O243' then O243=**1**; if rdiag8 eq 'O243' then O243=**1**;

if rdiag9 eq 'O243' then O243=**1**; if rdiag10 eq 'O243' then O243=**1**;

if sdiag1 eq 'O244' then O244=**1**; if sdiag2 eq 'O244' then O24=**1**;

if sdiag3 eq 'O244' then O244=**1**; if sdiag4 eq 'O244' then O24=**1**;

if sdiag5 eq 'O244' then O244=**1**; if sdiag6 eq 'O244' then O24=**1**;

if sdiag7 eq 'O244' then O244=**1**; if sdiag8 eq 'O244' then O24=**1**;

if sdiag9 eq 'O244' then O244=**1**; if sdiag10 eq 'O244' then O24=**1**;

if rdiag1 eq 'O244' then O244=**1**; if rdiag2 eq 'O244' then O244=**1**;

if rdiag3 eq 'O244' then O244=**1**; if rdiag4 eq 'O244' then O244=**1**;

if rdiag5 eq 'O244' then O244=**1**; if rdiag6 eq 'O244' then O244=**1**;

if rdiag7 eq 'O244' then O244=**1**; if rdiag8 eq 'O244' then O244=**1**;

if rdiag9 eq 'O244' then O244=**1**; if rdiag10 eq 'O244' then O244=**1**;

if sdiag1 eq 'O249' then O249=**1**; if sdiag2 eq 'O249' then O24=**1**;

if sdiag3 eq 'O249' then O249=**1**; if sdiag4 eq 'O249' then O24=**1**;

if sdiag5 eq 'O249' then O249=**1**; if sdiag6 eq 'O249' then O24=**1**;

if sdiag7 eq 'O249' then O249=**1**; if sdiag8 eq 'O249' then O24=**1**;

if sdiag9 eq 'O249' then O249=**1**; if sdiag10 eq 'O249' then O24=**1**;

if rdiag1 eq 'O249' then O249=**1**; if rdiag2 eq 'O249' then O249=**1**;

if rdiag3 eq 'O249' then O249=**1**; if rdiag4 eq 'O249' then O249=**1**;

if rdiag5 eq 'O249' then O249=**1**; if rdiag6 eq 'O249' then O249=**1**;

if rdiag7 eq 'O249' then O249=**1**; if rdiag8 eq 'O249' then O249=**1**;

if rdiag9 eq 'O249' then O249=**1**; if rdiag10 eq 'O249' then O249=**1**;

O24y=**100000***O240+**10000***O241+**1000***O242+**100***O243+**10***O244+**1***O249;

soseko2=**9**;

if soseko ge **30** and soseko le **39** then soseko2=**3**;

if soseko ge **40** and soseko le **49** then soseko2=**4**;

if soseko ge **50** and soseko le **59** then soseko2=**5**;

if soseko ge **1** and soseko le **5** then soseko2=**4**;

if soseko ge **6** and soseko le **9** then soseko2=**3**;

if aiemmatsynnytykset ge **4** then aiemmatsynnytykset=**4**;

if aiemmatsynnytykset eq **.** then aiemmatsynnytykset=**99**;

primi=**0**;

if aiemmatsynnytykset=**0** then primi=**1**;

aitikalk=**.**;

if aiti_ika lt **20** then aitikalk=**2**;

if aiti_ika ge **20** and aiti_ika le **24** then aitikalk=**3**;

if aiti_ika ge **25** and aiti_ika le **29** then aitikalk=**4**;

if aiti_ika ge **30** and aiti_ika le **34** then aitikalk=**5**;

if aiti_ika ge **35** and aiti_ika le **39** then aitikalk=**6**;

if aiti_ika ge **40** and aiti_ika le **44** then aitikalk=**7**;

if aiti_ika ge **45** and aiti_ika le **54** then aitikalk=**8**;

if aiti_ika eq **.** then aitikalk=**9**;

if apaino ge **150** then apaino=**.** ;

if apaino lt **10** then apaino= **.** ;

if apituus ge **200** then apituus=**.** ;

if apituus lt **100** then apituus=**.** ;

bmi=**10000***apaino/(apituus*apituus);

bmilk=**9**;

if bmi ge **10** and bmi lt **20** then bmilk=**1**;

if bmi ge **20** and bmi lt **25** then bmilk=**1**;

if bmi ge **25** and bmi lt **30** then bmilk=**2**;

if bmi ge **30** and bmi lt **40** then bmilk=**3**;

if bmi ge **40** then bmilk=**3**;

rkestvk=substr(kestovkpv,**1**,**2**);

ennena=**0**; if rkestvk ge **16** and rkestvk lt **37** then ennena=**1**;

pienip=**0**; if syntymapaino ge **200** and syntymapaino lt **2500** then pienip=**1**;

kuolema=**0**; if kuolleisuus eq **1** or kuolleisuus eq **2** then kuolema=**1**;

p90=**0**;

if sukup eq '1' and syntymapaino ge **586** and rkestvk eq **22** then p90=**1**;

if sukup eq '1' and syntymapaino ge **614** and rkestvk eq **23** then p90=**1**;

if sukup eq '1' and syntymapaino ge **809** and rkestvk eq **24** then p90=**1**;

if sukup eq '1' and syntymapaino ge **945** and rkestvk eq **25** then p90=**1**;

if sukup eq '1' and syntymapaino ge **1079** and rkestvk eq **26** then p90=**1**;

if sukup eq '1' and syntymapaino ge **1198** and rkestvk eq **27** then p90=**1**;

if sukup eq '1' and syntymapaino ge **1390** and rkestvk eq **28** then p90=**1**;

if sukup eq '1' and syntymapaino ge **1680** and rkestvk eq **29** then p90=**1**;

if sukup eq '1' and syntymapaino ge **1741** and rkestvk eq **30** then p90=**1**;

if sukup eq '1' and syntymapaino ge **2041** and rkestvk eq **31** then p90=**1**;

if sukup eq '1' and syntymapaino ge **2257** and rkestvk eq **32** then p90=**1**;

if sukup eq '1' and syntymapaino ge **2485** and rkestvk eq **33** then p90=**1**;

if sukup eq '1' and syntymapaino ge **2764** and rkestvk eq **34** then p90=**1**;

if sukup eq '1' and syntymapaino ge **3129** and rkestvk eq **35** then p90=**1**;

if sukup eq '1' and syntymapaino ge **3424** and rkestvk eq **36** then p90=**1**;

if sukup eq '1' and syntymapaino ge **3746** and rkestvk eq **37** then p90=**1**;

if sukup eq '1' and syntymapaino ge **3998** and rkestvk eq **38** then p90=**1**;

if sukup eq '1' and syntymapaino ge **4080** and rkestvk eq **39** then p90=**1**;

if sukup eq '1' and syntymapaino ge **4240** and rkestvk eq **40** then p90=**1**;

if sukup eq '1' and syntymapaino ge **4380** and rkestvk eq **41** then p90=**1**;

if sukup eq '1' and syntymapaino ge **4430** and rkestvk eq **42** then p90=**1**;

if sukup eq '1' and syntymapaino ge **4302** and rkestvk eq **43** then p90=**1**;

if sukup eq '2' and syntymapaino ge **510** and rkestvk eq **22** then p90=**1**;

if sukup eq '2' and syntymapaino ge **621** and rkestvk eq **23** then p90=**1**;

if sukup eq '2' and syntymapaino ge **741** and rkestvk eq **24** then p90=**1**;

if sukup eq '2' and syntymapaino ge **867** and rkestvk eq **25** then p90=**1**;

if sukup eq '2' and syntymapaino ge **966** and rkestvk eq **26** then p90=**1**;

if sukup eq '2' and syntymapaino ge **1380** and rkestvk eq **27** then p90=**1**;

if sukup eq '2' and syntymapaino ge **1442** and rkestvk eq **28** then p90=**1**;

if sukup eq '2' and syntymapaino ge **1540** and rkestvk eq **29** then p90=**1**;

if sukup eq '2' and syntymapaino ge **1684** and rkestvk eq **30** then p90=**1**;

if sukup eq '2' and syntymapaino ge **1987** and rkestvk eq **31** then p90=**1**;

if sukup eq '2' and syntymapaino ge **2196** and rkestvk eq **32** then p90=**1**;

if sukup eq '2' and syntymapaino ge **2448** and rkestvk eq **33** then p90=**1**;

if sukup eq '2' and syntymapaino ge **2590** and rkestvk eq **34** then p90=**1**;

if sukup eq '2' and syntymapaino ge **3096** and rkestvk eq **35** then p90=**1**;

if sukup eq '2' and syntymapaino ge **3330** and rkestvk eq **36** then p90=**1**;

if sukup eq '2' and syntymapaino ge **3610** and rkestvk eq **37** then p90=**1**;

if sukup eq '2' and syntymapaino ge **3835** and rkestvk eq **38** then p90=**1**;

if sukup eq '2' and syntymapaino ge **3965** and rkestvk eq **39** then p90=**1**;

if sukup eq '2' and syntymapaino ge **4080** and rkestvk eq **40** then p90=**1**;

if sukup eq '2' and syntymapaino ge **4206** and rkestvk eq **41** then p90=**1**;

if sukup eq '2' and syntymapaino ge **4333** and rkestvk eq **42** then p90=**1**;

if sukup eq '2' and syntymapaino ge **4194** and rkestvk eq **43** then p90=**1**;

sga=**2**;

if sukup eq '1' and rkestvk eq **24** and syntymapaino lt **527** then sga=**1**;

if sukup eq '1' and rkestvk eq **25** and syntymapaino lt **596** then sga=**1**;

if sukup eq '1' and rkestvk eq **26** and syntymapaino lt **679** then sga=**1**;

if sukup eq '1' and rkestvk eq **27** and syntymapaino lt **779** then sga=**1**;

if sukup eq '1' and rkestvk eq **28** and syntymapaino lt **896** then sga=**1**;

if sukup eq '1' and rkestvk eq **29** and syntymapaino lt **998** then sga=**1**;

if sukup eq '1' and rkestvk eq **30** and syntymapaino lt **1305** then sga=**1**;

if sukup eq '1' and rkestvk eq **31** and syntymapaino lt **1306** then sga=**1**;

if sukup eq '1' and rkestvk eq **32** and syntymapaino lt **1424** then sga=**1**;

if sukup eq '1' and rkestvk eq **33** and syntymapaino lt **1711** then sga=**1**;

if sukup eq '1' and rkestvk eq **34** and syntymapaino lt **1819** then sga=**1**;

if sukup eq '1' and rkestvk eq **35** and syntymapaino lt **2019** then sga=**1**;

if sukup eq '1' and rkestvk eq **36** and syntymapaino lt **2234** then sga=**1**;

if sukup eq '1' and rkestvk eq **37** and syntymapaino lt **2399** then sga=**1**;

if sukup eq '1' and rkestvk eq **38** and syntymapaino lt **2560** then sga=**1**;

if sukup eq '1' and rkestvk eq **39** and syntymapaino lt **2697** then sga=**1**;

if sukup eq '1' and rkestvk eq **40** and syntymapaino lt **2792** then sga=**1**;

if sukup eq '1' and rkestvk eq **41** and syntymapaino lt **2866** then sga=**1**;

if sukup eq '1' and rkestvk eq **42** and syntymapaino lt **2875** then sga=**1**;

if sukup eq '1' and rkestvk eq **43** and syntymapaino lt **2816** then sga=**1**;

if sukup eq '1' and rkestvk eq **24** and syntymapaino gt **878** then sga=**3**;

if sukup eq '1' and rkestvk eq **25** and syntymapaino gt **993** then sga=**3**;

if sukup eq '1' and rkestvk eq **26** and syntymapaino gt **1131** then sga=**3**;

if sukup eq '1' and rkestvk eq **27** and syntymapaino gt **1298** then sga=**3**;

if sukup eq '1' and rkestvk eq **28** and syntymapaino gt **1493** then sga=**3**;

if sukup eq '1' and rkestvk eq **29** and syntymapaino gt **1664** then sga=**3**;

if sukup eq '1' and rkestvk eq **30** and syntymapaino gt **2175** then sga=**3**;

if sukup eq '1' and rkestvk eq **31** and syntymapaino gt **2176** then sga=**3**;

if sukup eq '1' and rkestvk eq **32** and syntymapaino gt **2373** then sga=**3**;

if sukup eq '1' and rkestvk eq **33** and syntymapaino gt **2851** then sga=**3**;

if sukup eq '1' and rkestvk eq **34** and syntymapaino gt **3031** then sga=**3**;

if sukup eq '1' and rkestvk eq **35** and syntymapaino gt **3365** then sga=**3**;

if sukup eq '1' and rkestvk eq **36** and syntymapaino gt **3723** then sga=**3**;

if sukup eq '1' and rkestvk eq **37** and syntymapaino gt **3999** then sga=**3**;

if sukup eq '1' and rkestvk eq **38** and syntymapaino gt **4266** then sga=**3**;

if sukup eq '1' and rkestvk eq **39** and syntymapaino gt **4495** then sga=**3**;

if sukup eq '1' and rkestvk eq **40** and syntymapaino gt **4653** then sga=**3**;

if sukup eq '1' and rkestvk eq **41** and syntymapaino gt **4776** then sga=**3**;

if sukup eq '1' and rkestvk eq **42** and syntymapaino gt **4791** then sga=**3**;

if sukup eq '1' and rkestvk eq **43** and syntymapaino gt **4693** then sga=**3**;

if sukup eq '2' and rkestvk eq **24** and syntymapaino lt **527** then sga=**1**;

if sukup eq '2' and rkestvk eq **25** and syntymapaino lt **596** then sga=**1**;

if sukup eq '2' and rkestvk eq **26** and syntymapaino lt **679** then sga=**1**;

if sukup eq '2' and rkestvk eq **27** and syntymapaino lt **779** then sga=**1**;

if sukup eq '2' and rkestvk eq **28** and syntymapaino lt **896** then sga=**1**;

if sukup eq '2' and rkestvk eq **29** and syntymapaino lt **1040** then sga=**1**;

if sukup eq '2' and rkestvk eq **30** and syntymapaino lt **1122** then sga=**1**;

if sukup eq '2' and rkestvk eq **31** and syntymapaino lt **1349** then sga=**1**;

if sukup eq '2' and rkestvk eq **32** and syntymapaino lt **1440** then sga=**1**;

if sukup eq '2' and rkestvk eq **33** and syntymapaino lt **1605** then sga=**1**;

if sukup eq '2' and rkestvk eq **34** and syntymapaino lt **1742** then sga=**1**;

if sukup eq '2' and rkestvk eq **35** and syntymapaino lt **1978** then sga=**1**;

if sukup eq '2' and rkestvk eq **36** and syntymapaino lt **2165** then sga=**1**;

if sukup eq '2' and rkestvk eq **37** and syntymapaino lt **2326** then sga=**1**;

if sukup eq '2' and rkestvk eq **38** and syntymapaino lt **2463** then sga=**1**;

if sukup eq '2' and rkestvk eq **39** and syntymapaino lt **2591** then sga=**1**;

if sukup eq '2' and rkestvk eq **40** and syntymapaino lt **2687** then sga=**1**;

if sukup eq '2' and rkestvk eq **41** and syntymapaino lt **2757** then sga=**1**;

if sukup eq '2' and rkestvk eq **42** and syntymapaino lt **2764** then sga=**1**;

if sukup eq '2' and rkestvk eq **43** and syntymapaino lt **2645** then sga=**1**;

if sukup eq '2' and rkestvk eq **24** and syntymapaino gt **878** then sga=**3**;

if sukup eq '2' and rkestvk eq **25** and syntymapaino gt **993** then sga=**3**;

if sukup eq '2' and rkestvk eq **26** and syntymapaino gt **1131** then sga=**3**;

if sukup eq '2' and rkestvk eq **27** and syntymapaino gt **1298** then sga=**3**;

if sukup eq '2' and rkestvk eq **28** and syntymapaino gt **1376** then sga=**3**;

if sukup eq '2' and rkestvk eq **29** and syntymapaino gt **1734** then sga=**3**;

if sukup eq '2' and rkestvk eq **30** and syntymapaino gt **1870** then sga=**3**;

if sukup eq '2' and rkestvk eq **31** and syntymapaino gt **2249** then sga=**3**;

if sukup eq '2' and rkestvk eq **32** and syntymapaino gt **2400** then sga=**3**;

if sukup eq '2' and rkestvk eq **33** and syntymapaino gt **2675** then sga=**3**;

if sukup eq '2' and rkestvk eq **34** and syntymapaino gt **2904** then sga=**3**;

if sukup eq '2' and rkestvk eq **35** and syntymapaino gt **3296** then sga=**3**;

if sukup eq '2' and rkestvk eq **36** and syntymapaino gt **3609** then sga=**3**;

if sukup eq '2' and rkestvk eq **37** and syntymapaino gt **3876** then sga=**3**;

if sukup eq '2' and rkestvk eq **38** and syntymapaino gt **4105** then sga=**3**;

if sukup eq '2' and rkestvk eq **39** and syntymapaino gt **4318** then sga=**3**;

if sukup eq '2' and rkestvk eq **40** and syntymapaino gt **4478** then sga=**3**;

if sukup eq '2' and rkestvk eq **41** and syntymapaino gt **4595** then sga=**3**;

if sukup eq '2' and rkestvk eq **42** and syntymapaino gt **4606** then sga=**3**;

if sukup eq '2' and rkestvk eq **43** and syntymapaino gt **4408** then sga=**3**;

sga1=**1**; if sga eq **1** then sga1=**0**;

lga1=**1**; if sga eq **3** then lga1=**0**;

apgar5=**0**;

if apgar_5min eq '00' then apgar5=**1**; if apgar_5min eq '01' then apgar5=**1**;

if apgar_5min eq '02' then apgar5=**1**; if apgar_5min eq '03' then apgar5=**1**;

if apgar_5min eq '04' then apgar5=**1**; if apgar_5min eq '05' then apgar5=**1**;

if sokeri_patol eq '.' then sokeri_patol='0';

if insuliini_aloitettu eq '.' then insuliini_aloitettu='0';

if sokeri_tehty eq '0' then delete;

jako=**30**;

if (sokeri_patol eq '1' or insuliini_aloitettu eq '1' or O244 eq **1** or O249 eq **1**) and bmilk eq **1** then jako=**11**;

if (sokeri_patol eq '1' or insuliini_aloitettu eq '1' or O244 eq **1** or O249 eq **1**) and bmilk eq **2** then jako=**12**;

if (sokeri_patol eq '1' or insuliini_aloitettu eq '1' or O244 eq **1** or O249 eq **1**) and bmilk eq **3** then jako=**13**;

if (sokeri_patol eq '1' or insuliini_aloitettu eq '1' or O244 eq **1** or O249 eq **1**) and bmilk eq **9** then jako=**19**;

if sokeri_tehty eq '1' and sokeri_patol eq '0' and (insuliini_aloitettu eq '0' and O244 eq **0** and O249 eq **0**) and bmilk eq **1** then jako=**21**;

if sokeri_tehty eq '1' and sokeri_patol eq '0' and (insuliini_aloitettu eq '0' and O244 eq **0** and O249 eq **0**) and bmilk eq **2** then jako=**22**;

if sokeri_tehty eq '1' and sokeri_patol eq '0' and (insuliini_aloitettu eq '0' and O244 eq **0** and O249 eq **0**) and bmilk eq **3** then jako=**23**;

if sokeri_tehty eq '1' and sokeri_patol eq '0' and (insuliini_aloitettu eq '0' and O244 eq **0** and O249 eq **0**) and bmilk eq **9** then jako=**29**;

if sokeri_tehty eq '0' then jako=**90**;

if O240 eq **1** then jako=**99**;

if O241 eq **1** then jako=**99**;

if sokeri_tehty eq '0' then jako=**90**;

normal=**0**;

if sokeri_patol eq '1' then normal=**1**;

insulin=**0**;

if insuliini_aloitettu eq '1' then insulin=**1**;

rdb=**100**;

if O244 eq **1** then rdb=**200**; if O249 eq **1** then rdb=**200**;

yhteinen=rdb+jako;

lopullinen=**0**;

if jako eq **21** then lopullinen=**10**;

if jako eq **11** and insulin eq **0** then lopullinen=**11**;

if jako eq **11** and insulin eq **1** then lopullinen=**13**;

if jako eq **22** then lopullinen=**20**;

if jako eq **12** and insulin eq **0** then lopullinen=**21**;

if jako eq **12** and insulin eq **1** then lopullinen=**23**;

if jako eq **23** then lopullinen=**30**;

if jako eq **13** and insulin eq **0** then lopullinen=**31**;

if jako eq **13** and insulin eq **1** then lopullinen=**33**;

tehovalvonta=**0**;

if teho eq '1' then tehovalvonta=**1**;

if valvonta eq '1' then tehovalvonta=**1**;

if muusair eq '1' then tehovalvonta=**1**;

makros=**0**;

if syntymapaino ge **4000** and syntymapaino le **9000** then makros=**1**;

sektio=**0**;

if synnytystapatunnus eq '5' then sektio=**1**;

if synnytystapatunnus eq '6' then sektio=**1**;

if synnytystapatunnus eq '7' then sektio=**1**;

if synnytystapatunnus eq '8' then sektio=**1**;

ssektio=**0**;

if synnytystapatunnus eq '5' then ssektio=**1**;

muusektio=**0**;

if synnytystapatunnus eq '6' then muusektio=**1**;

if synnytystapatunnus eq '7' then muusektio=**1**;

if synnytystapatunnus eq '8' then muusektio=**1**;

P70=**0**; P704=**0**;

dg1=substr(ICD10_1,**1**,**3**); dg2=substr(ICD10_1,**1**,**3**); dg3=substr(ICD10_1,**1**,**3**);

dg4=substr(ICD10_4,**1**,**3**); dg5=substr(ICD10_1,**1**,**3**); dg6=substr(ICD10_1,**1**,**3**);

dg7=substr(ICD10_7,**1**,**3**); dg8=substr(ICD10_1,**1**,**3**); dg9=substr(ICD10_1,**1**,**3**);

dg10=substr(ICD10_10,**1**,**3**);

if dg1 eq 'P70' then P70=**1**; if dg2 eq 'P70' then P70=**1**; if dg3 eq 'P70' then P70=**1**;

if dg4 eq 'P70' then P70=**1**; if dg5 eq 'P70' then P70=**1**; if dg6 eq 'P70' then P70=**1**;

if dg7 eq 'P70' then P70=**1**; if dg8 eq 'P70' then P70=**1**; if dg9 eq 'P70' then P70=**1**;

if dg10 eq 'P70' then P70=**1**;

if ICD10_1 eq 'P704' then P704=**1**; if ICD10_2 eq 'P704' then P704=**1**; if ICD10_3 eq 'P704' then P704=**1**;

if ICD10_4 eq 'P704' then P704=**1**; if ICD10_5 eq 'P704' then P704=**1**; if ICD10_6 eq 'P704' then P704=**1**;

if ICD10_7 eq 'P704' then P704=**1**; if ICD10_8 eq 'P704' then P704=**1**; if ICD10_9 eq 'P704' then P704=**1**;

if ICD10_10 eq 'P704' then P704=**1**;

normal2=**0**; overw1=**0**; overw2=**0**; obes1=**0**; obes2=**0**;

if bmilk eq **1** and normal eq **1** then normal2=**1**;

if bmilk eq **2** and normal eq **0** then overw1=**1**;

if bmilk eq **2** and normal eq **1** then overw2=**1**;

if bmilk eq **3** and normal eq **0** then obes1=**1**;

if bmilk eq **3** and normal eq **1** then obes2=**1**;

normal3=**0**;

if bmilk eq **1** and normal eq **1** then normal3=**1**;

if bmilk eq **2** and normal eq **0** then normal3=**2**;

if bmilk eq **2** and normal eq **1** then normal3=**3**;

if bmilk eq **3** and normal eq **0** then normal3=**4**;

if bmilk eq **3** and normal eq **1** then normal3=**5**;

tupakka=**0**; if tupakointitunnus eq '2' then tupakka=**1**; if tupakointitunnus eq '3' then tupakka=**1**;

if bmilk eq **9** then delete;

uusijako=**9**;

if bmilk eq **1** and jako eq **11** or jako eq **30** then uusijako=**1**;

if bmilk eq **1** and jako eq **21** then uusijako=**0**;

if bmilk eq **2** and jako eq **12** or jako eq **30** then uusijako=**1**;

if bmilk eq **2** and jako eq **22** then uusijako=**0**;

if bmilk eq **3** and jako eq **13** or jako eq **30** then uusijako=**1**;

if bmilk eq **3** and jako eq **23** then uusijako=**0**;

normal3a=**0**; if bmilk eq **1** and uusijako eq **1** then normal3a=**1**;

normal3b=**0**; if bmilk eq **2** and uusijako eq **0** then normal3b=**1**;

normal3c=**0**; if bmilk eq **2** and uusijako eq **1** then normal3c=**1**;

normal3d=**0**; if bmilk eq **3** and uusijako eq **0** then normal3d=**1**;

normal3e=**0**; if bmilk eq **3** and uusijako eq **1** then normal3e=**1**;

if uusijako eq **9** then delete;

run;

**proc** **freq** data=peristat2;

table normal3 jako uusijako bmilk lopullinen

/nocol norow nopercent;

**run**;

**proc** **freq** data=peristat2;

table normal bmilk lopullinen ennena kaynnistys sektio makros apgar5 P704 tehovalvonta kuolema

soseko2 tupakka /nocol norow nopercent;

**run**;

**proc** **freq** data=peristat2;

table (AITI_IKA AIEMMATSYNNYTYKSET SOSEKO2 bmilk tupakka) * NORMAL /nocol norow nopercent all;

**run**;

**proc** **freq** data=peristat2;

table (AITI_IKA AIEMMATSYNNYTYKSET SOSEKO2 bmilk tupakka) * uusijako /nocol norow nopercent all;

**run**;

**proc** **freq** data=peristat2;

table bmilk * uusijako * (ssektio muusektio) /nocol norow nopercent all;

**run**;

**proc** **freq** data=peristat2;

table bmilk * uusijako * (tehovalvonta) /nocol norow nopercent all;

**run**;

**proc** **freq** data=peristat2;

table bmilk * uusijako/nocol norow nopercent all;

**run**;

**run**;

**proc** **freq** data=peristat2;

table jako * (LGA1 sektio ssektio muusektio tehovalvonta)/nocol norow nopercent all;

**run**;

**proc** **freq** data=peristat2;

table jako * (kaynnistys ennena apgar5 P70 kuolema)/nocol norow nopercent all;

**run**;

**proc** **logistic** data=PERISTAT2 descending;

model Sektio=AITI_IKA AIEMMATSYNNYTYKSET TUPAKKA SOSEKO2 normal3a normal3b normal3c normal3d normal3e

/risklimits;

**run**;

**proc** **logistic** data=PERISTAT2 descending;

model SSektio=AITI_IKA AIEMMATSYNNYTYKSET TUPAKKA SOSEKO2 normal3a normal3b normal3c normal3d normal3e

/risklimits;

**run**;

**proc** **logistic** data=PERISTAT2 descending;

model MUUSektio=AITI_IKA AIEMMATSYNNYTYKSET TUPAKKA SOSEKO2 normal3a normal3b normal3c normal3d normal3e

/risklimits;

**run**;

**proc** **logistic** data=PERISTAT2 ;

model LGA1=AITI_IKA AIEMMATSYNNYTYKSET TUPAKKA SOSEKO2 normal3a normal3b normal3c normal3d normal3e

/risklimits;

**run**;

**proc** **logistic** data=PERISTAT2 descending;

model TEHOVALVONTA=AITI_IKA AIEMMATSYNNYTYKSET TUPAKKA SOSEKO2 normal3a normal3b normal3c normal3d normal3e

/risklimits;

**run**;

**proc** **logistic** data=PERISTAT2 descending;

model ENNENA=AITI_IKA AIEMMATSYNNYTYKSET TUPAKKA SOSEKO2 normal3a normal3b normal3c normal3d normal3e

/risklimits;

**run**;

**proc** **logistic** data=PERISTAT2 descending;

model KAYNNISTYS=AITI_IKA AIEMMATSYNNYTYKSET TUPAKKA SOSEKO2 normal3a normal3b normal3c normal3d normal3e

/risklimits;

**run**;

**proc** **logistic** data=PERISTAT2 descending;

model APGAR5=AITI_IKA AIEMMATSYNNYTYKSET TUPAKKA SOSEKO2 normal3a normal3b normal3c normal3d normal3e

/risklimits;

**run**;

**proc** **logistic** data=PERISTAT2 descending;

model P70=AITI_IKA AIEMMATSYNNYTYKSET TUPAKKA SOSEKO2 normal3a normal3b normal3c normal3d normal3e

/risklimits;

**run**;

**proc** **logistic** data=PERISTAT2 descending;

model KUOLEMA=AITI_IKA AIEMMATSYNNYTYKSET TUPAKKA SOSEKO2 normal3a normal3b normal3c normal3d normal3e

/risklimits;

**run**;

**proc** **logistic** data=PERISTAT2 descending;

model TEHOVALVONTA=AITI_IKA AIEMMATSYNNYTYKSET TUPAKKA SOSEKO2 P704 normal3a normal3b normal3c normal3d normal3e

/risklimits;

**run**;

**proc** **logistic** data=PERISTAT2 descending;

model ENNENA=AITI_IKA AIEMMATSYNNYTYKSET TUPAKKA SOSEKO2 normal*bmilk

/risklimits;

**run**;

**proc** **logistic** data=PERISTAT2 descending;

model KAYNNISTYS=AITI_IKA AIEMMATSYNNYTYKSET TUPAKKA SOSEKO2 normal*bmilk

/risklimits;

**run**;

**proc** **logistic** data=PERISTAT2 descending;

model Sektio=AITI_IKA AIEMMATSYNNYTYKSET TUPAKKA SOSEKO2 normal*bmilk

/risklimits;

**run**;

**proc** **logistic** data=PERISTAT2 descending;

model makros=AITI_IKA AIEMMATSYNNYTYKSET TUPAKKA SOSEKO2 normal*bmilk

/risklimits;

**run**;

**proc** **logistic** data=PERISTAT2 descending;

model APGAR5=AITI_IKA AIEMMATSYNNYTYKSET TUPAKKA SOSEKO2 normal*bmilk

/risklimits;

**run**;

**proc** **logistic** data=PERISTAT2 descending;

model p70=AITI_IKA AIEMMATSYNNYTYKSET TUPAKKA SOSEKO2 normal*bmilk

/risklimits;

**run**;

**proc** **logistic** data=PERISTAT2 descending;

model TEHOVALVONTA=AITI_IKA AIEMMATSYNNYTYKSET TUPAKKA SOSEKO2 normal*bmilk

/risklimits;

**run**;

**proc** **logistic** data=PERISTAT2 descending;

model KUOLEMA=AITI_IKA AIEMMATSYNNYTYKSET TUPAKKA SOSEKO2 normal*bmilk

/risklimits;

**run**;
